# Parallel Factorization to Implement Group Analysis in Brain Networks Estimation

**DOI:** 10.3390/s23031693

**Published:** 2023-02-03

**Authors:** Andrea Ranieri, Floriana Pichiorri, Emma Colamarino, Valeria de Seta, Donatella Mattia, Jlenia Toppi

**Affiliations:** 1 Department of Computer, Control and Management Engineering, Sapienza University of Rome, Via Ariosto, 25, 00185 Rome, Italy; 2 Neuroelectrical Imaging and Brain Computer Interface Lab, IRCCS Fondazione Santa Lucia, 00179 Rome, Italy

**Keywords:** connectivity estimation, tensor decomposition, parallel factorization, group analysis, EEG, partial directed coherence

## Abstract

When dealing with complex functional brain networks, group analysis still represents an open issue. In this paper, we investigated the potential of an innovative approach based on PARAllel FActorization (PARAFAC) for the extraction of the grand average connectivity matrices from both simulated and real datasets. The PARAFAC approach was solved using three different numbers of rank-one tensors (PAR-FACT). Synthetic data were parametrized according to different levels of three parameters: network dimension (NODES), number of observations (SAMPLE-SIZE), and noise (SWAP-CON) in order to investigate the way they affect the grand average estimation. PARAFAC was then tested on a real connectivity dataset, derived from EEG data of 17 healthy subjects performing wrist extension with left and right hand separately. Findings on both synthetic and real data revealed the potential of the PARAFAC algorithm as a useful tool for grand average extraction. As expected, the best performances in terms of FPR, FNR, and AUC were achieved for great values of sample size and low noise level. A crucial role has been revealed for the PAR-FACT parameter, revealing that an increase in the number of rank-one tensors solving the PARAFAC problem leads to an increase in FPR values and, thus, to a worse grand average estimation.

## 1. Introduction

Investigating the functional mechanisms underlying the complexity of the human brain still represents a challenging issue in modern biomedicine. The representation of the central nervous system as a complex network made its own way in traditional neuroscience, being validated at both structural and functional levels by anatomical studies and noninvasive neuroimaging techniques [1]. An analytical approach is thus required to deal with both identification and quantification of the existing connections within brain structures [2].

Brain networks can be derived from anatomical or physiological observations, resulting in structural and functional networks, respectively [3]. While structural connectivity describes how the different neural elements are anatomically connected, functional connectivity identifies statistical dependences among them [4,5]. Different techniques allow the estimation of such functional connections, at both the scalp and whole-brain level [6]; here, we focused on partial directed coherence (PDC), a spectral estimator relying on the concept of Granger causality [7], which determines the directed influence between a pair of signals in a multivariate data set. With respect to pairwise approaches, PDC allows us to distinguish between direct and cascade causal effects, reducing the number of false positives, as well as offering high accuracy, stability, and robustness to noise [8]. Depending on the definition of all multivariate autoregressive models, PDC includes all known (and accessible) sources of the problem into the model, thus avoiding spurious connections usually encountered within pairwise approaches. This approach does not require any a priori hypothesis about the underlying model and can thus be used when no information about the network structure is available.

Group level characterization of brain networks can give useful insights on the behavior of such a complex network within a given population. Group analysis can be used in neuroscience to investigate specific physiological or pathological conditions or to determine the existence of a common pattern, that is, the grand average, elicited by the same experimental paradigm in different subjects’ brain circuits. Such a problem implicitly requires an effective way to integrate information from multiple subjects, as well as handling the intrinsic inter-subject variability. 

Many different approaches have been proposed over the years, but there is currently no consensus on a univocal analysis pipeline for the extraction of the grand average connectivity pattern. In [9] Toppi et al. grand average connectivity patterns were estimated by comparing two different experimental conditions (i.e., task vs. baseline or task1 vs. task 2) at single-connection level applying the *t*-test across subjects. Grand average connections were identified by means of their *p*-value: a connection exists if the *p*-value associated to it in the statistical comparison is below the significance threshold. Since statistical comparison was repeated for each connection in the network, a family-wise error rate correction was applied in order to reduce type I errors due to multiple comparisons [10]. In fact, since the number of comparisons quadratically increases with the number of nodes, large networks often requires severe alpha level corrections: this is especially true when the Bonferroni correction is applied [11], resulting in extremely sparse matrices that can be difficult to interpret.

Pester et al. [12] proposed the use of the PARAllel FACtorisation (PARAFAC) algorithm to extract the grand average from fMRI derived networks in a group of subjects. Under the hypotheses of linearity, such approach allows for splitting the original data matrix into a sum of rank-1 tensors (i.e., factors), expressed as the outer product of a proper number of vectors. In this way, different connectivity patterns “hidden” in the original one can be found depending on the number of required factors. Each subject-specific network was properly rearranged as a first order tensor, and the whole cohort of patients was then collected into an integrative tensor for PARAFAC decomposition. The final sum of rank-one tensors can be expressed in terms of subject-independent and subject-dependent loadings: the first are translated into connectivity patterns, while the latter are used to find the most expressed pattern within the population.

Especially in the last decades, the PARAFAC algorithm proved to be a useful tool for the analysis of multidimensional biomedical data. In [13], Miwakeichi and colleagues decomposed a multichannel time-varying EEG spectrum into a series of distinct components, by means of a PARAFAC decomposition in space, frequency, and time domain. Furthermore, Spyrou et al. [14] exploited the ability of PARAFAC2 (an extension of PARAFAC) to deal with a complex tensor factorization of EEG into scalp components described by spatial, spectral and complex trial profiles. Combining the PARAFAC algorithm together with hierarchical clustering, Belyaeva et al. [15] proposed a tensor-based approach for the extraction of developmental signatures from multi-subject MEG data. They were able to extract early and late latency event-related field components, allowing for discrimination between high and low performance groups.

In this work, we investigated the potential of a PARAFAC-based approach to extract the grand average from both synthetic and real connectivity datasets. Synthetic data mimic PDC matrices of different subjects under the same experimental condition, parametrized according to different levels of (i) number of subjects in the group (SAMPLE-SIZE), (ii) inter-subject variability (SWAP-CON), and (iii) number of nodes (NODES). The way the number of required factors for PARAFAC application (PAR-FACT) affects the final performance was also investigated. The algorithm was then tested on a real dataset made by PDC matrices of 17 healthy subjects. Connectivity matrices were extracted from EEG signals recorded during the execution of the left- and right-hand extension, separately.

## 2. Materials and Methods

### 2.1. Partial Directed Coherence

When dealing with autoregressive representation of multivariate time series, Partial Directed Coherence (PDC) [16] was demonstrated to be a frequency domain description of Granger causality between time series [7]. Consider Y as a set of time series:

(1) $$Y\left(t\right)=\left[y_{1}\left(t\right),\;y_{2}\left(t\right),\;\ldots ,\;y_{K}\left(t\right)\right]$$

where t refers to time and Y(t) is made up of K number of signals. A proper description for Y(t) by means of the following multivariate autoregressive (MVAR) model is as follows:

(2) $$\overset{P}{\underset{p=0}{\sum }}\lambda \left(p\right)Y\left(t-p\right)=\varepsilon \left(t\right),\;with\;\;\lambda \left(0\right)=I$$

where $\varepsilon \left(t\right)={\left[\varepsilon_{1}\left(t\right),\ldots ,\varepsilon_{K}\left(t\right)\right]}^{T}$ is a vector of zero-mean uncorrelated white noise processes, λ(1),λ(2),…,λ(P) are the K×K matrices containing model’s coefficients, and p is the model order, usually chosen by means of the Akaike information criteria (AIC) for MVAR processes [17]. By applying the Fourier Transform on both, Equation (2) turns into:

(3) Λ(f)Y(f)=E(f)

where the convolution product on the left side of Equation (2) has been transformed into the product Λ(f)Y(f). The term Λ(f) represents the Fourier transform along the p lags of the parameters vector λ(p). Each element of Λ(f) can be expressed as:

(4) $$\Lambda_{ij}\left(f\right)=\delta_{ij}-\overset{P}{\underset{p=1}{\sum }}\lambda_{ij}\left(p\right)e^{-sqrt\left(-1\right)2\pi fp}$$

where sqrt (−1) indicates the imaginary unit and $\delta_{ij}=1$ whenever i=j and $\delta_{ij}=0$ otherwise. Λ(f) appears in the definition of PDC directed from signal j to signal i as follows:

(5) $$\pi_{ij}\left(f\right)=\frac{{\left|\Lambda_{ij}\left(f\right)\right|}^{2}}{\sum_{m=1}^{K}{\left|\Lambda_{mj}\left(f\right)\right|}^{2}}$$

Squared versions of PDC in its different normalizations are usually adopted, due to higher stability and accuracy [18].

### 2.2. Tensor Decomposition by Means of Parallel factorization (PARAFAC)

A tensor is a multidimensional array. More formally, an $N^{th}$ order tensor is an element of the tensor product of N vector spaces, each of which has its own coordinate system. Since it is not easy to manipulate an N-dimensional tensor, different techniques have been developed over the years in order to properly split its original informative content into a lower-dimension easier to handle mathematical objects. In such a scenario, tensor decomposition techniques have been used to extract knowledge from high-dimensional tensors in many different fields, from physics to machine learning

PARAFAC is a decomposition technique that factorizes an $N^{th}$ order tensor into a sum of R rank one tensors, where R is the rank of the original tensor [19]. According to tensorial linear algebra principles, an $N^{th}$ order tensor is rank one if it can be rewritten as the outer product of N vectors:

(6) $$X=a^{\left(1\right)}⊗a^{\left(2\right)}⊗\ldots \;⊗a^{\left(N\right)}$$

where the symbol “⊗” represents the vector outer product. It follows that:

(7) $$X=a⊗b=ab^{T}=\left[\begin{array}{cccc} a_{1}b_{1} & a_{1}b_{2} & \cdots & \;a_{1}b_{M}\\ \;a_{2}b_{1} & \;a_{2}b_{2} & \cdots & a_{2}b_{M}\\ \vdots & \vdots & \cdots & \vdots \\ a_{N}b_{1} & a_{N}b_{2}\; & \cdots & a_{N}b_{M} \end{array}\right],\;X\in R^{N\times M}.\;a\in R^{N},\;b\in R^{M}$$

As a consequence, the PARAFAC decomposition of a rank R third order tensor $X\in R^{I\times J\times D}$ can be rewritten as:

(8) $$X=\overset{R}{\underset{r=1}{\sum }}X_{r}+E=\overset{R}{\underset{r=1}{\sum }}a^{\left(r\right)}⊗b^{\left(r\right)}⊗c^{\left(r\right)}+E$$

where $a\in R^{I},\;b\in R^{J},\;c\in R^{D}$, and $E\in R^{I\times J\times D}$ is a residual error term. Since R is the rank of the original tensor, PARAFAC decomposition of the original dataset contains up to R rank-one tensors whose linear combination gives the original information in X. By setting the number of required rank-one tensors to any 1≤f≤R, the original informative content of X can be split into an arbitrary (but bounded) number of factors. Elementwise, Equation (8) can be rewritten as

(9) $$x_{ijk}=\overset{R}{\underset{r=1}{\sum }}a_{i}{}^{\left(r\right)}b_{j}{}^{\left(r\right)}c_{d}{}^{\left(r\right)}+e_{ijd}$$

Each term $a_{i}{}^{\left(r\right)}$, $b_{j}{}^{\left(r\right)}$, and $c_{d}{}^{\left(r\right)}$ in Equation (9) acts as a weight expressing the contribution of the $r^{th}$ component of a,  b, and c on $x_{ijd}$. A compact matrix representation can be obtained by stacking the $a^{\left(r\right)}$ vectors side by side, defining the columns of the $A\in R^{I\times R}$ matrix.

(10) $$A=\left[\begin{array}{ccc} a_{1}{}^{\left(1\right)} & \cdots & a_{1}{}^{\left(R\right)}\\ \vdots & \ddots & \vdots \\ a_{I}{}^{\left(1\right)} & \cdots & a_{I}{}^{\left(R\right)} \end{array}\right]$$

Repeating for the $b^{\left(r\right)}$ and $c^{\left(r\right)}$ leads to the construction of matrices $B\in R^{J\times R}$ and $C\in R^{D\times R}$, which are a compact representation of vectors $b^{\left(r\right)}$ and $c^{\left(r\right)}$ for r=1, 2,…, R.

By switching the error term to the left side of Equation (8), $\hat{X}=X-E$ can be considered as an approximation of the original tensor X: in such a way, the classic PARAFAC algorithm finds out the best $\hat{X}$ that minimizes the sum of squares of the residuals $e_{ijd}$ [20]. For the sake of simplicity, consider a third-order tensor X: its PARAFAC decomposition can be obtained by solving the following optimization problem.

(11) $$\left|\left|\;X-\hat{X}\;\right|\right|\;\;\mathrm{with}\;\hat{X}=\sum_{r=1}^{R}a^{\left(r\right)}⊗b^{\left(r\right)}⊗c^{\left(r\right)}$$

The solution to the problem in Equation (11) can be found by means of the alternating least squares (ALS) method. The key idea is to fix all factor matrices except for one in order to optimize for the non-fixed matrix and then repeat this step for every matrix, until some stopping criterion is satisfied [21]. If an estimate of B and C is given, the Khatri-Rao product between $B\in R^{J\times R}$ and $C\in R^{D\times R}$ produces the matrix $B⊙C\in R^{\left(IJ\right)\times R}$ defined as

(12) $$B⊙C=\left[b^{\left(1\right)}⊗c^{\left(1\right)}\;b^{\left(2\right)}⊗c^{\left(2\right)}\;\ldots \;b^{\left(R\right)}⊗c^{\left(R\right)}\;\right]$$

Matricization allows for properly rearranging the elements of an $N^{th}$ order tensor into a matrix. The mode-n matricization of a tensor $X\in R^{I_{1}\times I_{2}\times \ldots \times I_{N}}$, denoted by $X_{\left(n\right)}$, arranges the mode-n fibers to be the column of the resulting matrix [19]. By letting Z=B⊙C, the matrix A can be determined by solving the following optimization problem

(13) $$\left|\left|\;X_{\left(1\right)}-AZ^{T}\right|\right|$$

which has a solution in the least square sense given by

(14) $$A=X_{\left(1\right)}Z^{†}=X_{\left(1\right)}{\left(B⊙C\right)}^{†}$$

where $Z^{†}=Z^{T}{\left(ZZ^{T}\right)}^{-1}$ is the pseudoinverse Moore–Penrose matrix. In such a way, for the third order tensor the ALS algorithm would perform the following steps repeatedly until convergence.

(15) $$A\;\leftarrow \left|\left|\;X_{\left(1\right)}-\left(B⊙C\right)A^{T}\right|\right|\;B\;\leftarrow \left|\left|\;X_{\left(2\right)}-\left(A⊙C\right)B^{T}\right|\right|\;C\;\leftarrow \left|\left|\;X_{\left(3\right)}-\left(A⊙B\right)C^{T}\right|\right|$$

### 2.3. PARAFAC to Extract the Grand Average Connectivity Matrix

A generic connectivity dataset X obtained from a population of individuals is composed by s different K x K connectivity matrices, where s is the number of individuals and K is the number of nodes. Each subject’s connectivity pattern can be properly rearranged as a first order tensor $\pi^{\left(s\right)}\in R^{K^{2}}$, with s=1, 2…S. In such a way, PARAFAC algorithm can then be applied to the second order tensor $X\in R^{K^{2}\times S}$, whose columns represent each subject’s vectorized connectivity pattern $\pi^{\left(s\right)}$ for s=1, 2…S:

(16) $$X=\left[\begin{array}{cccc} \pi_{11}^{\left(1\right)} & \pi_{11}^{\left(2\right)} & \cdots & \pi_{11}^{\left(S\right)}\\ \pi_{12}^{\left(1\right)} & \pi_{12}^{\left(2\right)} & \ldots & \pi_{12}^{\left(S\right)}\\ \vdots & \vdots & \ldots & \vdots \\ \pi_{KK}^{\left(1\right)} & \pi_{KK}^{\left(2\right)} & \cdots & \pi_{KK}^{\left(S\right)} \end{array}\right]=\left[\pi^{\left(1\right)}\pi^{\left(2\right)}\;\ldots \pi^{\left(S\right)}\;\right]$$

where $\pi_{ij}^{\left(s\right)}$ represents the $ij^{th}$ entry of PDC matrix of subject s. Since $X\in R^{K^{2}\times S}$, the number of linearly independent column of X is necessarly smaller, or at least equal, to S; this naturally impose an upper boundary to the number of factors to be set in PARAFAC algorithm:

(17) $$\{\begin{array}{c} R=rank\left(X\right)\leq S\\ f\leq R=rank\left(X\right) \end{array}\;,\;necessarly\;\to f\leq S$$

Since X is a bidimensional tensor, it can be decomposed by means of PARAFAC algorithm as

(18) $$X=\overset{f}{\underset{r=1}{\sum }}a^{\left(r\right)}⊗b^{\left(r\right)}+E$$

or, elementwise

(19) $$x_{ij}=\overset{f}{\underset{r=1}{\sum }}a_{i}{}^{\left(r\right)}b_{j}{}^{\left(r\right)}+e_{ij}$$

The terms $a_{i}{}^{\left(r\right)}$ (respectively $b_{j}{}^{\left(r\right)}$) indicate the way the $i^{th}$ element of the vector $a^{\left(r\right)}$ (respectively the $j^{th}$ element of $b^{\left(r\right)}$) modulates $x_{ij}$. In this kind of scenario $a_{i}$ represents the strength of a specific PDC connection, while $b_{j}$ are the subject-wise loadings indicating how relevant is the PDC pattern in $a^{\left(r\right)}$ for the reconstruction of X [5].

In order to identify the grand average pattern ${\tilde{a}}^{\left(r\right)}$ that better describes the overall behavior within the population, we looked for the column ${\tilde{b}}^{\left(r\right)}$ of $B\in R^{s\times f}$ with minimum variance across subjects. In fact, since if $a^{\left(r\right)}$ is a recurrent PDC pattern in X, it is expected to be equally expressed in a large number of individuals. This means that the elements of the correspondent subject-wise loading vector $b_{j}{}^{\left(r\right)}$ are expected to be very similar to each other.

For the purpose of this study, the minimization problem in Equation (11) has been substituted by

(20) $$\left|\;X-\hat{X}\;\right|\;\;\mathrm{with}\;\hat{X}=\sum_{r=1}^{f}a^{\left(r\right)}⊗b^{\left(r\right)}$$

where the Euclidean norm was substituted by the L1-norm. In such a way, instead of minimizing the sum of squares of the residuals, we looked for the $a^{\left(r\right)}$ and $b^{\left(r\right)}$ that minimize the sum of the residuals. We preferred this kind of approach since it produces a sparse matrix $\hat{X}$ which better suits the interpretation of PDC pattern, with respect to a full matrix obtained with the classic problem in Equation (11).

### 2.4. Testing PARAFAC Algorithm on Synthetic Data

PARAFAC algorithm was tested on different synthetic datasets representing a set of connectivity matrices obtained from a group of participants for a specific experimental condition. 

The simulation study included T=100 iterations of the following steps:

1. Generation of synthetic datasets composed of different connectivity matrices (one per subject) obtained as modified versions of a predefined ground-truth matrix representing the grand average. The percentage of modification was modulated in the study and the ground-truth was changed at each repetition of the simulation process;
2. Application of the PARAFAC algorithm to synthetic datasets in order to factorize the whole data matrix as a sum of rank-one tensors (see Section 2.3 for further details). The algorithm was applied using three values for the PARAFAC factors number (PAR-FACT: f={2, 5, 10});
3. Selection of the rank-one tensor that better represents the grand average connectivity pattern within the whole dataset;
4. Evaluation of the performances by comparing the rank-one tensor chosen at point 3 with the current ground-truth.

Analysis of variance (ANOVA) was then carried out to evaluate PARAFAC performances in terms of false positive and false negative rates (see Section 2.4.2 below for details) across the 100 iterations.

#### 2.4.1. Synthetic Data Generation

Synthetic data have been generated as a set of predefined individual binary connectivity matrices (one per subject), each obtained as randomly modified version of a predefined ground-truth matrix. Modifications of the original ground-truth matrix consisted in the displacement of connections between different positions in the matrix. The density of the network was fixed to 0.1. In order to investigate both validity and robustness of the reconstructed patterns, synthetic data analysis has been parametrized with respect to different variables that could variably affect the decomposition approach. These parameters were:

- dataset sample size (SAMPLE-SIZE, s∈{10, 20, 30, 50, 100}) defined as the number of subjects simulated in the group dataset;
- dataset dimension defined as the number of nodes of the simulated connectivity matrices (NODES, k∈{20, 30, 50});
- percentage of swapped connections with respect to the original ground-truth network (SWAP-CON, m∈{0.10, 0.30, 0.50}) representing different levels of inter-subject variability of the connectivity matrices in the group (noise level).

#### 2.4.2. Performance Evaluation

In dealing with the identification of the grand average connectivity pattern, PARAFAC accuracy was quantified by means of false positive and false negative rates. The comparison between the extracted rank-one tensor representing the grand average and the imposed ground-truth network provided four different parameters [22]:

- False Positives (FP), defined as the total number of null connections in the ground truth labeled as not-null in the grand average;
- True Positives (TP) defined as the total number of not-null connections in both the grand average and ground truth;
- False Negatives (FP), defined as the total number of not-null connections in the ground truth labeled as null in the grand average;
- True Negatives (TN) defined as the total number of null connections in both the grand average and ground truth.

For the evaluation of PARAFAC performances, we used:

(21) $$FPR=\frac{FP}{FP+TN}$$

(22) $$FNR=\frac{FN}{TP+FN}$$

In order to have a synthetic measure including both parameters, we also considered the Area Under the receiver operating characteristic Curve (AUC) [23].

#### 2.4.3. Statistical Analysis

Three separate three-way repeated measures Analyses of Variance (ANOVAs) were applied considering as main within factors SAMPLE-SIZE (s∈{10, 20, 30, 50, 100}), SWAP-CON, (m∈{0.10, 0.30, 0.50}) and PAR-FACT (f∈{2, 5, 10}) and as dependent variables the three performance parameters (FPR, FNR and AUC), separately. The same ANOVAs were repeated for each number of nodes considered in the study (NODES, k∈{20, 30, 50}). The statistical significance level for all tests was set to 0.05, and the Tukey’s post-hoc test was performed to assess differences among the levels of the within factors.

### 2.5. Testing PARAFAC Algorithm on Real EEG Data

#### 2.5.1. Participants

The testing of PARAFAC algorithm on real data was carried out on EEG data recorded from 17 healthy subjects (mean age: 48±17 years, 11 females/6 males, all right-handed except one) during the execution of simple hand motor tasks at the Laboratory of Neuroelectrical Imaging and Brain Computer Interface of Fondazione Santa Lucia, IRCCS, Rome, Italy. The study was approved by the local ethics board at Fondazione Santa Lucia, IRCCS, Rome, Italy (CE PROG.752/2019), the protocol was written according to the Helsinki Declaration, and all the participants signed an informed consent.

#### 2.5.2. Experimental Design

Scalp EEG potentials were collected with a sampling frequency of 1 kHz from 61 active electrodes arranged according to an extension of 10–20 system (reference on left mastoid and ground on right mastoid) by means of BrainAmp amplifiers (Brain Products GmbH, 82205 Gilching, Germany), impedances were kept below 5 kΩ.

EEG data have been acquired according to the experimental protocol in [24]. Each participant was asked to sit in a comfortable chair while visual cues were presented on a screen in front of him/her via Matlab’s Psychtoolbox. Each experimental session consisted of four runs (with a break between each of them) in which patients were asked to perform finger extension (Ext) and grasping (Grasp) with the left and right hand separately. Each run comprised 40 trials equally divided in task (8s duration) and rest (4s duration) condition presented to the participants in a pseudo-random order. Task trials began with 4s of a preparatory period, after which a go stimulus occurred and the participant were asked to perform the required task for 4s. In rest trials, instead, participants were asked to stay relaxed for the whole trial duration. A fixation cross was displayed for 3s in the middle of the screen during each inter-trial interval.

#### 2.5.3. EEG Data Processing

The acquired EEG data were bandpass filtered [3−60] Hz) and a notch filter at 50 Hz was applied to remove power-line artifacts. Ocular artifacts were removed by means of independent component analysis. Task trials were segmented in the window [5−7] s from the cue onset and classified according to the two tasks (Ext_L, Ext_R). It was, in fact, reasonable to hypothesize, in such window, the steady-state condition for motor task and thus the breakdown of transient phenomena [25]. Residual artifacts (e.g., muscular, environmental) were lastly removed using a semiautomatic procedure based on the use of a threshold criterion (±100 µV).

From the original acquisition setup, 24 out of 61 electrodes (FC5, FC3, FC1, FC2, FC4, FC6, C5, C3, C1, C2, C4, C6, CP5, CP3, CP1, CP2, CP4, CP6, P5, P3, P1, P2, P4, and P6) were selected in order to put the focus on brain areas usually engaged during motor tasks (i.e., sensorimotor areas from centro-frontal to centro-parietal areas). For each subject and experimental condition, we estimated connectivity matrices by means of PDC estimator in four frequency bands (α [8−12] Hz, β [13−30] Hz, γ [31−40] Hz, and θ [3−7] Hz). Matrices were binarized by means the asymptotic statistics approach allowing us to discard spurious connections from the null-case [22,26]. In particular, we set to 1 all the connections whose value was above its corresponding theoretical null-case threshold and to 0 all the connections whose value was below the corresponding threshold. 

Before applying PARAFAC decomposition, a spatial filter was set in order to remove the 8-neighbours short-distance connections between adjacent electrodes (except between sagittal lines 1 and 2). This step allows us to remove input spurious connections due to volume–conduction phenomena, which will corrupt the final estimated pattern. The binarized PDC patterns of the overall subjects, obtained for extension task (Ext_L, Ext_R) and frequency bands, were properly arranged in a data matrix $X\in R^{K^{2}\times S}$ (with N=24 and S=17) and then given as input to the PARAFAC algorithm separately. The approach was repeated for two different values of PAR-FACT parameter (f={2,5}). As for synthetic data, for each task and band, we selected the rank-one tensor characterized by the lower level of variance in subjects’ loadings as the one representing the grand average. Such tensor was then binarized setting to 0 all the values below 0.001 and re-arranged as a K×K connectivity matrix. The value of each non-null connection provided by the algorithm has been substituted with the mean PDC value of that specific connection within the population. All these steps allowed us to identify a grand average connectivity matrix for each task and frequency band.

#### 2.5.4. Performance Evaluations by Means of Degree Scalp Maps

When considering the human brain as a complex network, degree distribution offers useful insight in describing the role of different brain areas. Since information flow within the brain is bidirectional, for each node v both output and input connections have to be considered. Degree of a specific node v in the network (deg(v)) is defined as the sum of incoming (id(v)) and outcoming (od(v)) connections of that node:

(23) deg(v)=id(v)+od(v)

As shown in Equation (23), when dealing with adjacency matrices, the joint degree distribution is nothing but the sum of output and input degree of each vertex [27]. Such a distribution often provides a strong indicator of influence or centrality of different brain areas. By means of scalp maps, the join degree distribution allows us to quantitatively appreciate the role of each electrode with respect to the current network pattern. 

## 3. Results

### 3.1. Performances of PARAFAC Algorithm on Synthetic Data

For each NODES level, a 3-way repeated measure ANOVA was carried out on FPR, FNR, and AUC separately, with SAMPLE-SIZE, SWAP-CON, and PAR-FACT as main within factors. In Table 1 we reported the results of the ANOVA conducted on FPR. We noticed how either single or combined factors have a significant effect on FPR distribution. 

**Figure 1:**
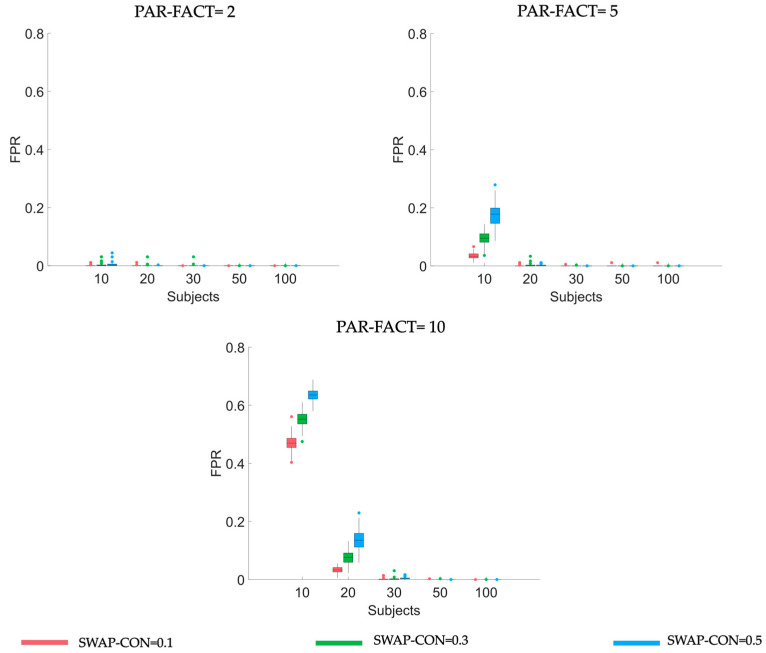
Boxplot diagrams reporting FPR distributions obtained applying PARAFAC algorithm using three different numbers of PAR-FACT (panels along rows in the figure) on synthetic data generated using five different levels of SAMPLE-SIZE (along x-axis), different levels of SWAP-CON (different colors of the bars). Results refer to synthetic datasets of dimension equal to 20 nodes.

In order to describe such an effect, in Figure 1 we reported FPR distributions obtained applying the PARAFAC algorithm for different numbers of PAR-FACT to connectivity matrices of a dimension equal to 20 nodes and characterized by different sample-sizes (SAMPLE-SIZE) and different levels of noise (SWAP-CON). It is worth noting how FPR values became worse in response to an increase in the number of factors set in the PARAFAC algorithm or in the percentage of swapped connections (increase of noise corruption), when sample size is low (i.e., s<20). Using two factors led to the FPR close to zero for all the sample sizes and percentages of swapped connections. When the PAR-FACT = 10, the FPR varies from 50 to 70% when SAMPLE-SIZE = 10 and from 5 to 15% when SAMPLE-SIZE = 20, depending on the noise level. The number of involved subjects has a mitigating effect, reducing FPR values as the cohort of recruited patients increases. This was true for all the numbers of PARAFAC factors and all the percentages of noise corruption. FPR went to zero for a sample size higher than 30. Similar results were found for all NODES levels (see Supplementary Materials for results related to K=30, 50).

**Figure 2:**
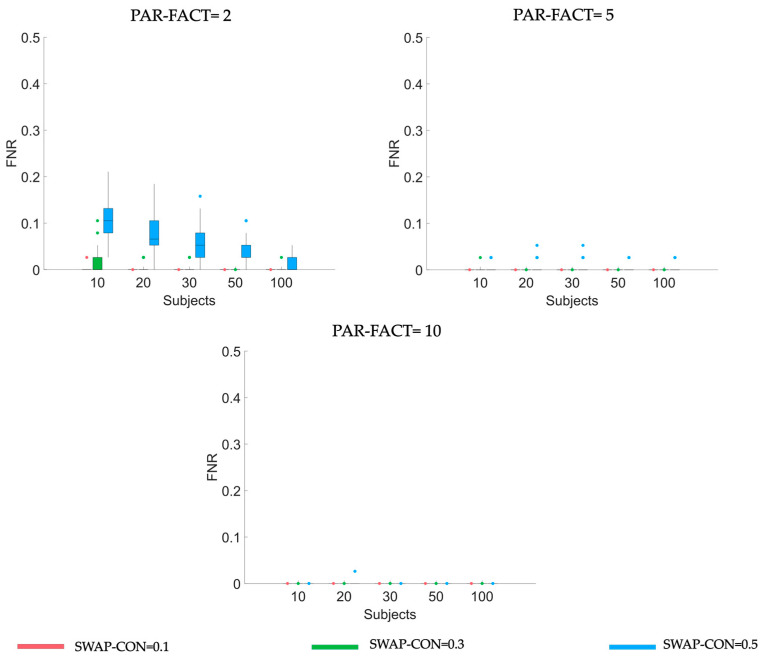
Boxplot diagrams reporting FNR distributions obtained applying PARAFAC algorithm using three different numbers of PAR-FACT (panels along rows in the figure) on synthetic data generated using five different levels of SAMPLE-SIZE (along x-axis), different levels of SWAP-CON (different colors of the bars). Results refer to synthetic datasets of dimension equal to 20 nodes.

Results of the 3-way repeated measures ANOVA on FNR are summarized in Table 2. As for FPR, either single or combined ANOVA variables significantly affect the FNR distribution. For each f in PAR-FACT levels, boxplots in Figure 2 illustrate how FNR distribution changes according to different levels of the generating variables (NODES, SWAP-CON, and SAMPLE-SIZE). Diagrams allow us to appreciate that FNR values are close to zero when f>2, regardless of SAMPLE-SIZE, SWAP-CON, or NODES level. Instead, when PAR-FACT = 2, FNR decreases with an increase in SAMPLE-SIZE or a decrease in SWAP-CON, as confirmed by Tukey’s post-hoc test. Similar considerations were found for all NODES levels (see Supplementary Materials).

**Figure 3:**
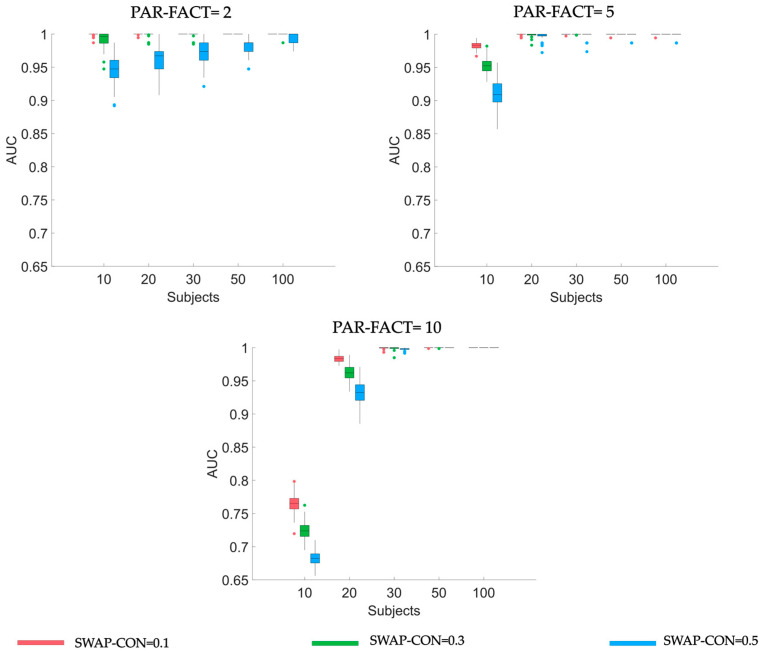
Boxplot diagrams reporting AUC distributions obtained applying PARAFAC algorithm using three different numbers of PAR-FACT (panels along rows in the figure) on synthetic data generated using five different levels of SAMPLE-SIZE (along x-axis), different levels of SWAP-CON (different colors of the bars). Results refer to synthetic datasets of dimension equal to 20 nodes.

Table 3 reported results of the 3-way repeated measures ANOVA on AUC values for different numbers of nodes. According to boxplot representation in Figure 3, AUC values are close to 1 (i.e., optimal classification of null and non-null connections) for s>20 without regards to NODES, SWAP-CON, or PAR-FACT level. When SAMPLE-SIZE is lower than 20, strong differences exist between the 2- and 10-factors decomposition. In fact, as highlighted by Tukey’s post-hoc test, when PAR-FACT = 2 AUC values are significantly higher (ranging from 0.95 to 1) than those obtained when PAR-FACT = 10 (ranging from 0.68 to 0.78) for all SWAP-CON levels. However, as expected, AUC values decrease when SWAP-CON level increases. Similar results were found for all NODES levels (see Supplementary Materials).

### 3.2. Performance of PARAFAC Algorithm on Real EEG Data

**Figure 4:**
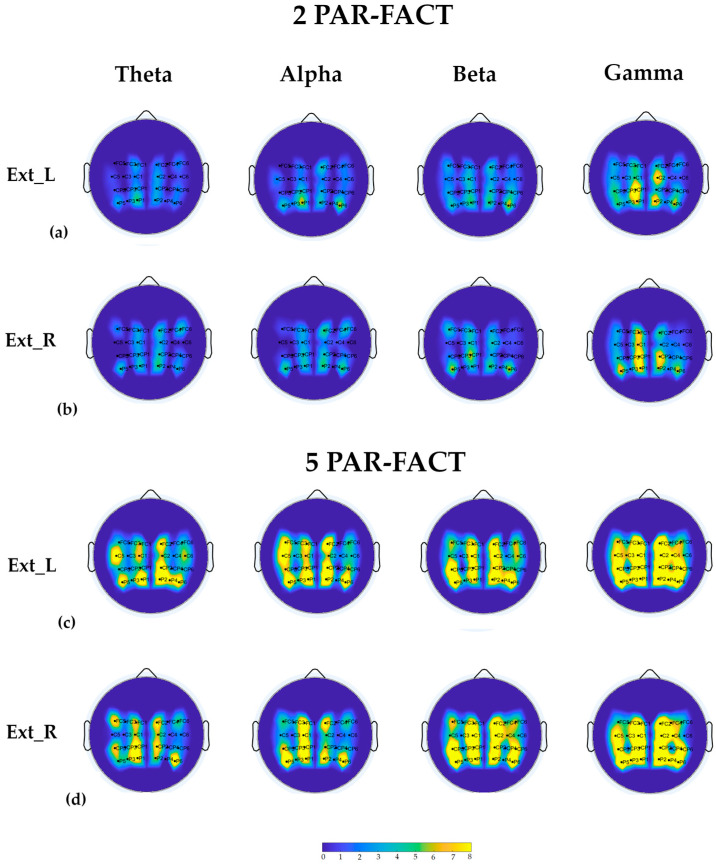
Degree scalp maps obtained from the GA connectivity matrices estimated applying PARAFAC algorithm with 2 (panels (**a**,**b**)) and 5 (panels (**c**,**d**)) PAR-FACT in two different tasks (extension of left hand–panels (**a**,**c**), extension of right hand–panels (**b**,**d**)) for the four frequency bands.

PARAFAC algorithm was applied to the EEG dataset collected in a group of 17 healthy subjects during the execution of wrist extension with left and right hand separately. The extraction of PDC matrices for each task allowed for brain connectivity estimation in four different frequency bands (α, β, γ , and θ ). Adjacency matrices have then been extracted for each band by means of asymptotic thresholding [28]. Grand average connectivity matrices were then obtained for each frequency band considering two different number of PAR-FACT (2 and 5 factors). The degree distribution was then calculated for each electrode, in order to provide a quantitative measure about the involvement of each node within the brain network. Figure 4 reported degree scalp maps obtained for the two different PARAFAC decomposition in the upper-mentioned frequency bands and for the two different tasks.

PARAFAC decomposition on real data confirmed what synthetic data analysis pointed out: increasing the number of PAR-FACT from 2 to 5 lead to different grand average estimations. In particular, using 5 led to an increase in the number of false positives, testified by the fact that almost all the electrodes showed the same high degree. As showed in Figure 4 (panels c and d), no involvement of specific scalp areas is evident: degree scalp maps showed a general activation of all the included electrodes.

Instead, the use of two factors allowed us to discriminate the role of different scalp areas during the execution of each task. Electrodes on sagittal lines close to the imaginary conjunction between inion and nasion (i.e., FC1, C1, CP1, P1, FC2, C2, CP2, and P2) showed the highest degree. Electrodes in the hemisphere contralateral to the moved hand showed, in general, a greater degree.

## 4. Discussion

In this work we exploited the potentiality of a PARAFAC-based approach to extract the common mode pattern from both synthetic and real connectivity datasets.

For synthetic data, ANOVA tables and boxplot representations in Section 3.1 revealed that either single or combined factors (SAMPLE-SIZE, SWAP-CON, or PAR-FACT) significantly affect the final decomposition and the estimated grand average pattern.

As expected, noise corruption and sample size have opposite effects: low levels of noise allowed for better estimation of the grand average pattern, whereas the opposite occurs for the sample size. Considering the noise corruption as the effect of the inter-subject variability and the number of subjects as the number of realizations of a noisy stochastic process, the search for the grand average pattern can be interpreted as the calculation of the “expected” pattern within the population. Because noise is assumed to be uncorrelated from the signal of interest (white process), under linearity assumptions the expected value for the sum between signal and noise is nothing but the summation of the expected value for the signal and the expected value for the noise. The expected value for a white noise process approaches zero as the number of realizations of such a process approaches the infinitive, thus justifying results about the effects of sample size on the estimated common pattern.

These considerations specifically hold when the effect of the inter-subject variability can be modeled as the superimposition of white noise to the grand average network. This is only true when the inter-subject variability is due to random processes related to experimental and technical factors. Depending on the situation, the inter-subject variability could be better represented as a variation in cognitive strategies carried on by the individuals or an activation of different brain circuits in response to the same cognitive task. In such a scenario, PARAFAC decomposition is not successful and thus advanced modeling procedures should be applied. The idea could be to set up a multisubject network [29] or to consider the set of single-subject networks as a multi-layer network ensemble with nodes connected via interlayer links [30].

Our findings also point out that the number of required factors significantly affects the grand average estimation. When dealing with data modeling, different empirical approaches have been proposed to determine the proper number of factors for the PARAFAC model that better suits the original dataset [31,32,33]. These approaches can be seen as a solution for blind source separation problems applied to EEG data. In most of the cases, the number of sources equals the number of channels used to record the original signal (e.g., several dozens) in order to deal with the intrinsic complexity of the EEG signal, as in the case of Independent Component Analysis, since the aim is to reproduce the high complexity of a multi-channel EEG dataset. However, in this manuscript, we proposed an alternative way to use PARAFAC. Since we are interested in the identification of an average behavior across subjects, the best way to proceed is to set the lowest number of PAR-FACT in order to identify the pattern in common among the subjects included in the analysis.

It has been empirically proved that when the number of required factors draw close to the rank of the original data matrix, each rank-one tensor approximates the specific pattern of each subject [12]. In the limit case for f=rank(X)=S, each factor (together with a residual error term) is expected to reflect the PDC pattern of each subject within the dataset, thus diverging from our purpose to look for the common mode. Furthermore, when orthogonality constraint is required for loadings [34], not each pattern nor its generating loadings are associated with any other pattern in the remaining components or their respective loadings. Since we only required the non-negativity of each factor, as f approaches rank(X) we expect each rank-one tensor to reflect just the singularities characterizing each subject’s pattern, being the common mode split on f different non-negative factors because of linearity assumption. In the ideal (unrealistic) situation in which all the subjects show the same connectivity pattern, we don’t need any decomposition, since the single pattern coincides with the common mode. By selecting two factors we assume that the first rank-one tensor represents the common mode, while the second one embodies a random pattern due to inter-subject variability.

Findings on synthetic data lead us to investigate both validity and robustness of the PARAFAC algorithm on a real dataset. As expected, the use of a greater number of factors led to an increase in the number of false positives. In fact, five-factor scalp maps show no involvement of specific scalp areas, but a general activation of all the included electrodes. Two-factor scalp maps allow the discrimination of the role of different scalp areas in the execution of the two motor tasks. The extracted grand average patterns show a higher degree on central and fronto-central lines electrodes mainly located in the hemisphere contralateral to the moved hand, which is expected according to the physiological EEG activity/reactivity during motor tasks which involves mainly electrodes above the contralateral sensorimotor cortex [35]. 

## 5. Conclusions

Although it has been widely used for data fitting and tensor decomposition problems, this is the first time, to the best of our knowledge, in which PARAFAC has been used in EEG-based functional connectivity context. In particular, we systematically investigated PARAFAC performances in the reconstruction of a grand average pattern from a set of connectivity matrices estimated in a group of subjects. The study conducted on simulated data, properly modulated according to factors typical of experimental studies in the network science field (number of nodes, number of subjects, noise level), not only demonstrated the brilliant performances of PARAFAC in group analysis, but also provided guidelines for its application in different contexts. In fact, the results obtained would help other researchers to set a PARAFAC number of factors and to define the appropriate sample-size of their studies in order to obtain such high performances.

In this paper, we applied PARAFAC algorithm to networks obtained by means of PDC estimator; however, the results could likely be extended to other connectivity approaches, and not only those related to the causality concept. As further development, we suggest comparing PARAFAC to other tensor decomposition techniques or, more generally, to different methods (e.g., Artificial Neural Networks, clustering techniques and Principal Component Analysis) to find the best approach for this purpose. Future works should also assess PARAFAC performances on real data in different neuroscience contexts since in this work we limited the investigation to networks underlying simple motor tasks.

## Figures and Tables

**Table 1:** Results of three-way repeated measures ANOVA computed considering FPR as dependent variable and PAR-FACT, SWAP-CON, and SAMPLE-SIZE as within main factors. The analysis was repeated for three different number of nodes (20, 30, 50). Acronym DOF is for degrees of freedom.

Effect	DOF	FPR 20 Nodes		FPR 30 Nodes		FPR 50 Nodes	
		F	*p*	F	*p*	F	*p*
PAR-FACT	(2, 198)	**50497.55**	**<0.01**	**72505.4**	**<0.01**	**80901.2**	**<0.01**
SWAP-CON	(2, 198)	**2200.03**	**<0.01**	**4011.2**	**<0.01**	**6098.7**	**<0.01**
SAMPLE-SIZE	(4, 396)	**65646.26**	**<0.01**	**83402.3**	**<0.01**	**94681.9**	**<0.01**
PAR-FACT * SWAP-CON	(4, 396)	**728.99**	**<0.01**	**1227.3**	**<0.01**	**2503.7**	**<0.01**
PAR-FACT * SAMPLE-SIZE	(8, 792)	**36742.33**	**<0.01**	**47493.4**	**<0.01**	**50507.5**	**<0.01**
SWAP-CON * SAMPLE-SIZE	(8, 792)	**1153.50**	**<0.01**	**1848.0**	**<0.01**	**2727.5**	**<0.01**
PAR-FACT * SWAP-CON * SAMPLE-SIZE	(16, 1584)	**381.90**	**<0.01**	**654.8**	**<0.01**	**1148.2**	**<0.01**

**Table 2:** Results of three-way repeated measures ANOVA computed considering FNR as dependent variable and PAR-FACT, SWAP-CON, and SAMPLE-SIZE as within main factors. The analysis was repeated for three different number of nodes (20, 30, 50). Acronym DOF is for degrees of freedom.

Effect	DOF	FNR 20 Nodes		FNR 30 Nodes		FNR 50 Nodes	
		F	*p*	F	*p*	F	*p*
PAR-FACT	(2, 198)	**1154.440**	**<0.01**	**3077.845**	**<0.01**	**6507.522**	**<0.01**
SWAP-CON	(2, 198)	**1156.072**	**<0.01**	**2580.131**	**<0.01**	**5589.628**	**<0.01**
SAMPLE-SIZE	(4, 396)	**111.501**	**<0.01**	**372.461**	**<0.01**	**720.475**	**<0.01**
PAR-FACT * SWAP-CON	(4, 396)	**1111.953**	**<0.01**	**2315.958**	**<0.01**	**5166.622**	**<0.01**
PAR-FACT * SAMPLE-SIZE	(8, 792)	**111.838**	**<0.01**	**377.123**	**<0.01**	**718.188**	**<0.01**
SWAP-CON * SAMPLE-SIZE	(8, 792)	**82.582**	**<0.01**	**235.413**	**<0.01**	**468.317**	**<0.01**
PAR-FACT * SWAP-CON * SAMPLE-SIZE	(16, 1584)	**79.142**	**<0.01**	**230.926**	**<0.01**	**456.722**	**<0.01**

**Table 3:** Results of three-way repeated measures ANOVA computed considering AUC as dependent variable and PAR-FACT, SWAP-CON, and SAMPLE-SIZE as within main factors. The analysis was repeated for three different number of nodes (20, 30, 50). Acronym DOF is for degrees of freedom.

Effect	DOF	AUC 20 Nodes		AUC 30 Nodes		AUC 50 Nodes	
		F	*p*	F	*p*	F	*p*
PAR-FACT	(2, 198)	**21577**	**<0.01**	**33271**	**<0.01**	**44600**	**<0.01**
SWAP-CON	(2, 198)	**2926**	**<0.01**	**7704**	**<0.01**	**10606**	**<0.01**
SAMPLE-SIZE	(4, 396)	**32272**	**<0.01**	**53239**	**<0.01**	**75013**	**<0.01**
PAR-FACT * SWAP-CON	(4, 396)	**174**	**<0.01**	**313**	**<0.01**	**830**	**<0.01**
PAR-FACT * SAMPLE-SIZE	(8, 792)	**15927**	**<0.01**	**26991**	**<0.01**	**31879**	**<0.01**
SWAP-CON * SAMPLE-SIZE	(8, 792)	**780**	**<0.01**	**1614**	**<0.01**	**2874**	**<0.01**
PAR-FACT * SWAP-CON * SAMPLE-SIZE	(16, 1584)	**103**	**<0.01**	**198**	**<0.01**	**489**	**<0.01**

## Data Availability

The data presented in this study may be available on reasonable request from the corresponding author. The data are not publicly available due to ethical and privacy restrictions.

## Supplementary Materials

The following supporting information can be downloaded at: https://www.mdpi.com/article/10.3390/s23031693/s1, Figure S1: Boxplot diagrams reporting FPR distributions obtained applying PARAFAC algorithm using three different numbers of PAR-FACT (panels along rows in the figure) on synthetic data generated using five different levels of SAMPLE-SIZE (along x-axis), different levels of SWAP-CON (different colors of the bars). Results refer to synthetic datasets of dimension equal to 30 nodes; Figure S2: Boxplot diagrams reporting FNR distributions obtained applying PARAFAC algorithm using three different numbers of PAR-FACT (panels along rows in the figure) on synthetic data generated using five different levels of SAMPLE-SIZE (along x-axis), different levels of SWAP-CON (different colors of the bars). Results refer to synthetic datasets of dimension equal to 30 nodes; Figure S3: Boxplot diagrams reporting AUC distributions obtained applying PARAFAC algorithm using three different numbers of PAR-FACT (panels along rows in the figure) on synthetic data generated using five different levels of SAMPLE-SIZE (along x-axis), different levels of SWAP-CON (different colors of the bars). Results refer to synthetic datasets of dimension equal to 30 nodes; Figure S4: Boxplot diagrams reporting FPR distributions obtained applying PARAFAC algorithm using three different numbers of PAR-FACT (panels along rows in the figure) on synthetic data generated using five different levels of SAMPLE-SIZE (along x-axis), different levels of SWAP-CON (different colors of the bars). Results refer to synthetic datasets of dimension equal to 50 nodes; Figure S5: Boxplot diagrams reporting FNR distributions obtained applying PARAFAC algorithm using three different numbers of PAR-FACT (panels along rows in the figure) on synthetic data generated using five different levels of SAMPLE-SIZE (along x-axis), different levels of SWAP-CON (different colors of the bars). Results refer to synthetic datasets of dimension equal to 50 nodes; Figure S6: Boxplot diagrams reporting AUC distributions obtained applying PARAFAC algorithm using three different numbers of PAR-FACT (panels along rows in the figure) on synthetic data generated using five different levels of SAMPLE-SIZE (along x-axis), different levels of SWAP-CON (different colors of the bars). Results refer to synthetic datasets of dimension equal to 50 nodes.
