# Supplementary material for: Parallel Factorization to Implement Group Analysis in Brain Networks Estimation
Source: Sensors (Basel). 2023 Feb 3;23(3):1693. doi: 10.3390/s23031693 (PMC9920099; doi:10.3390/s23031693)
Supplement: Supplementary file 1 [file sensors-23-01693-s001.zip › sensors-2145267-supplementary.pdf]

sensors

Parallel factorization to implement group analysis in brain networks estimation

Andrea Ranieri[1,2], Floriana Pichiorri[2], Emma Colamarino[1,2], Valeria de Seta[1,2], Donatella Mattia[2], Jlenia Toppi[1,2*]

[1] Dept. Of Computer, Control and Management Engineering, Sapienza University of Rome, Rome, Italy
[2] Neuroelectrical Imaging and Brain Computer Interface Lab, IRCCS Fondazione Santa Lucia, Rome, Italy
* Correspondence: jlenia.toppi@uniroma1.it; Tel.: +390651501165; Mail address: Via Ariosto, 25, 00185 Rome, Italy

Supplementary Materials

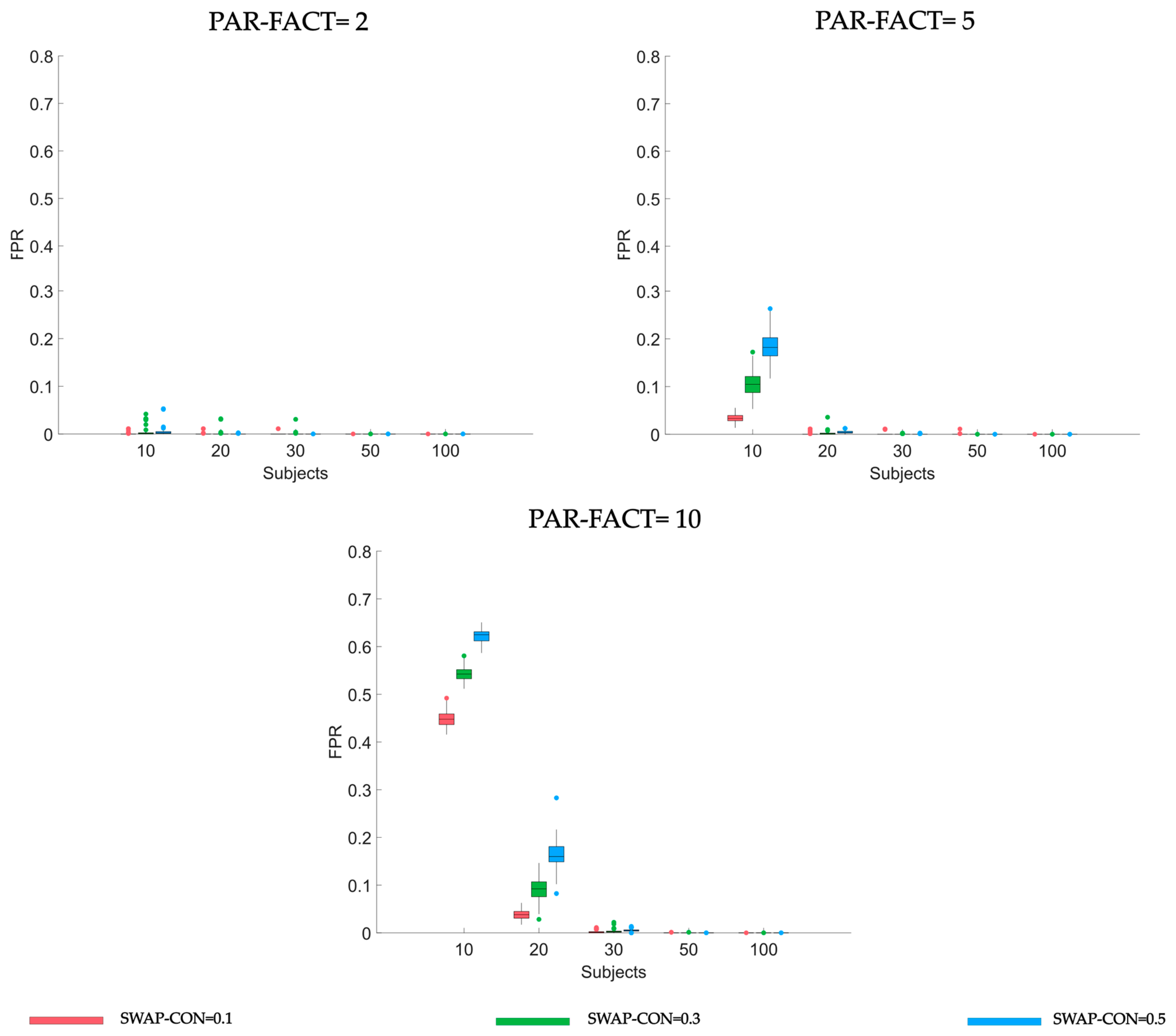

Figure S1. Boxplot diagrams reporting FPR distributions obtained applying PARAFAC algorithm using three different numbers of PAR-FACT (panels along rows in the figure) on synthetic data generated using five different levels of SAMPLE-SIZE (along x-axis), different levels of SWAP-CON (different colors of the bars). Results refer to synthetic datasets of dimension equal to 30 nodes.

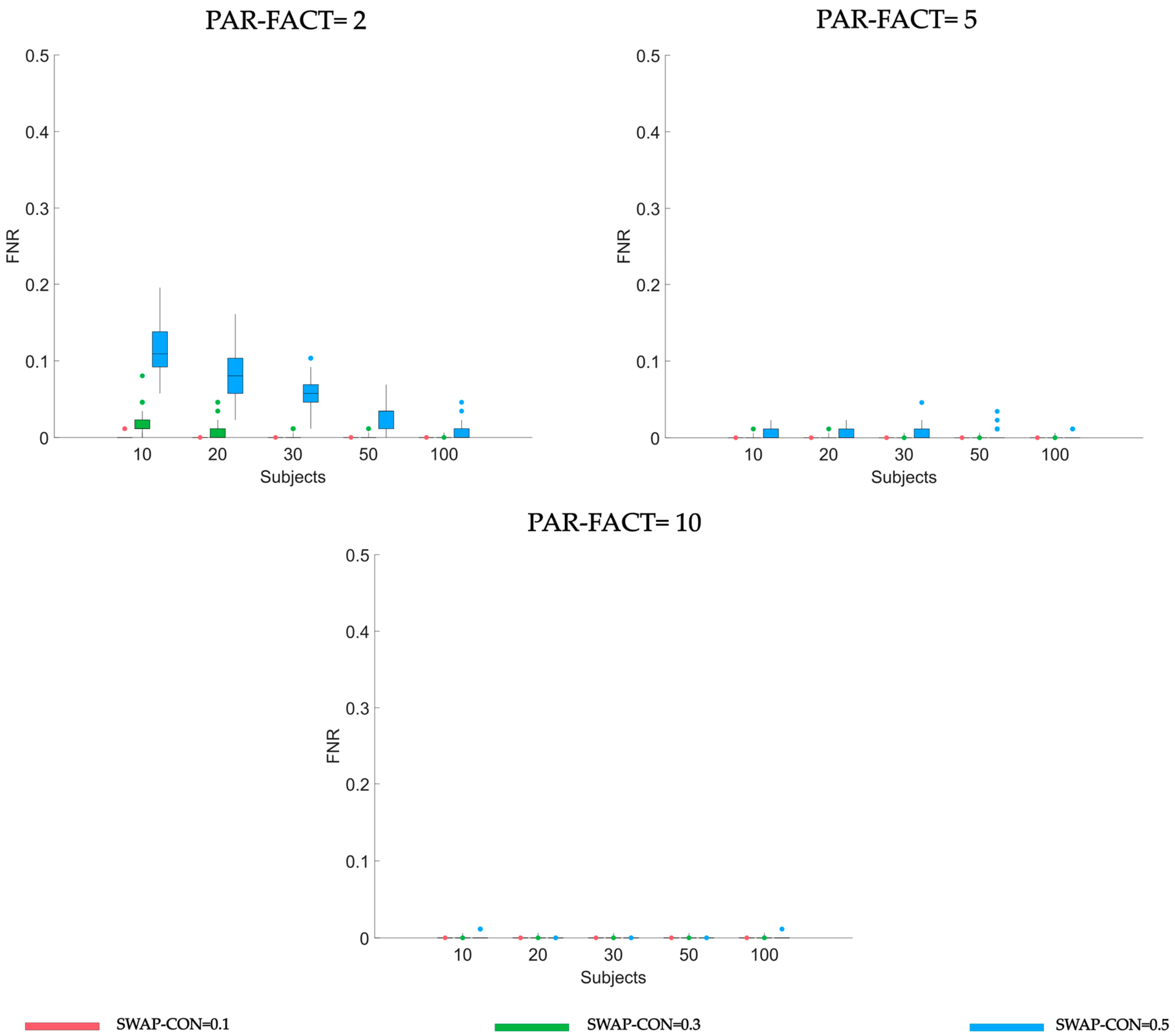

Figure S2. Boxplot diagrams reporting FNR distributions obtained applying PARAFAC algorithm using three different numbers of PAR-FACT (panels along rows in the figure) on synthetic data generated using five different levels of SAMPLE-SIZE (along x-axis), different levels of SWAP-CON (different colors of the bars). Results refer to synthetic datasets of dimension equal to 30 nodes.

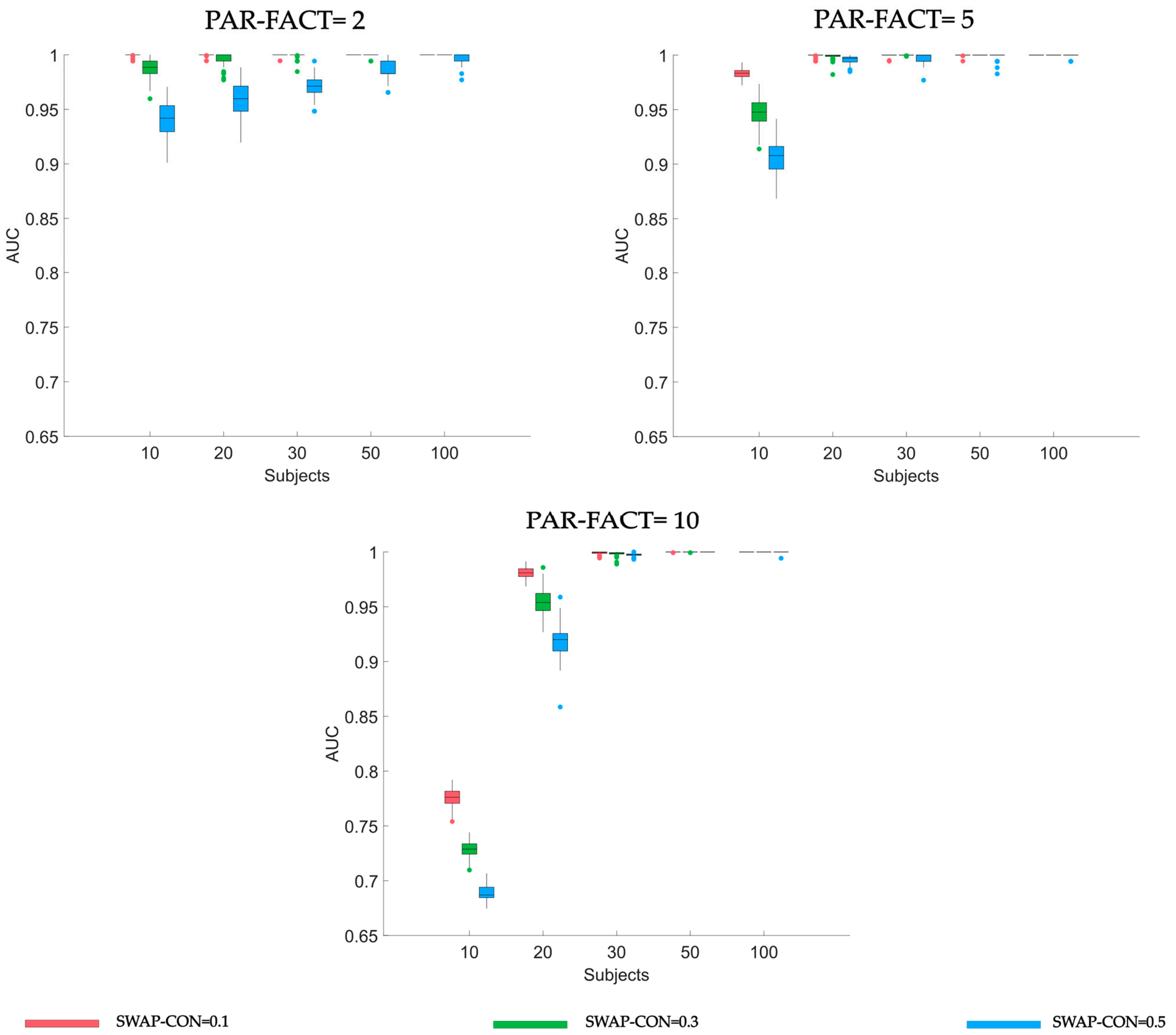

Figure S3. Boxplot diagrams reporting AUC distributions obtained applying PARAFAC algorithm using three different numbers of PAR-FACT (panels along rows in the figure) on synthetic data generated using five different levels of SAMPLE-SIZE (along x-axis), different levels of SWAP-CON (different colors of the bars). Results refer to synthetic datasets of dimension equal to 30 nodes.

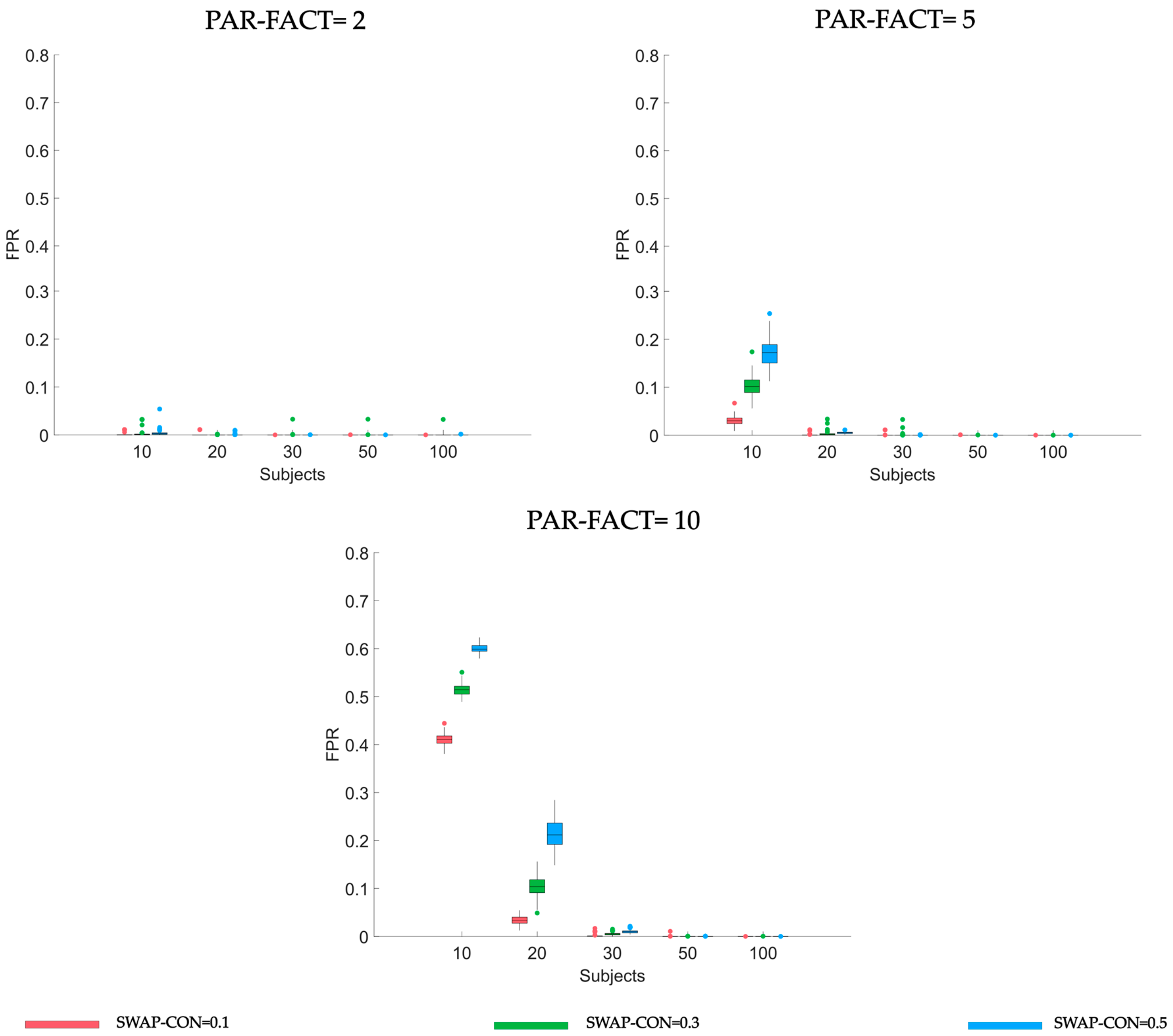

Figure S4. Boxplot diagrams reporting FPR distributions obtained applying PARAFAC algorithm using three different numbers of PAR-FACT (panels along rows in the figure) on synthetic data generated using five different levels of SAMPLE-SIZE (along x-axis), different levels of SWAP-CON (different colors of the bars). Results refer to synthetic datasets of dimension equal to 50 nodes.

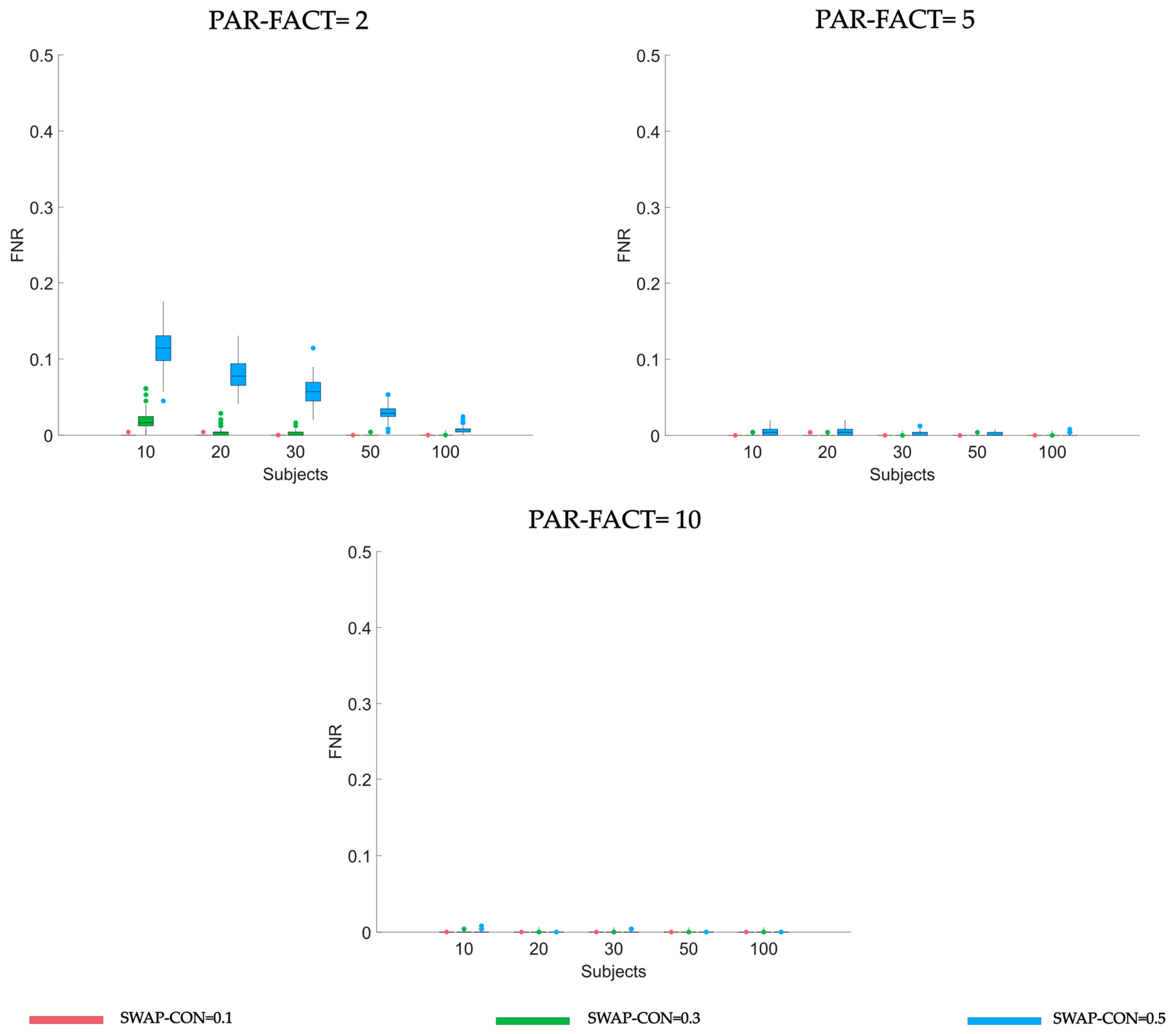

Figure S5. Boxplot diagrams reporting FNR distributions obtained applying PARAFAC algorithm using three different numbers of PAR-FACT (panels along rows in the figure) on synthetic data generated using five different levels of SAMPLE-SIZE (along x-axis), different levels of SWAP-CON (different colors of the bars). Results refer to synthetic datasets of dimension equal to 50 nodes.

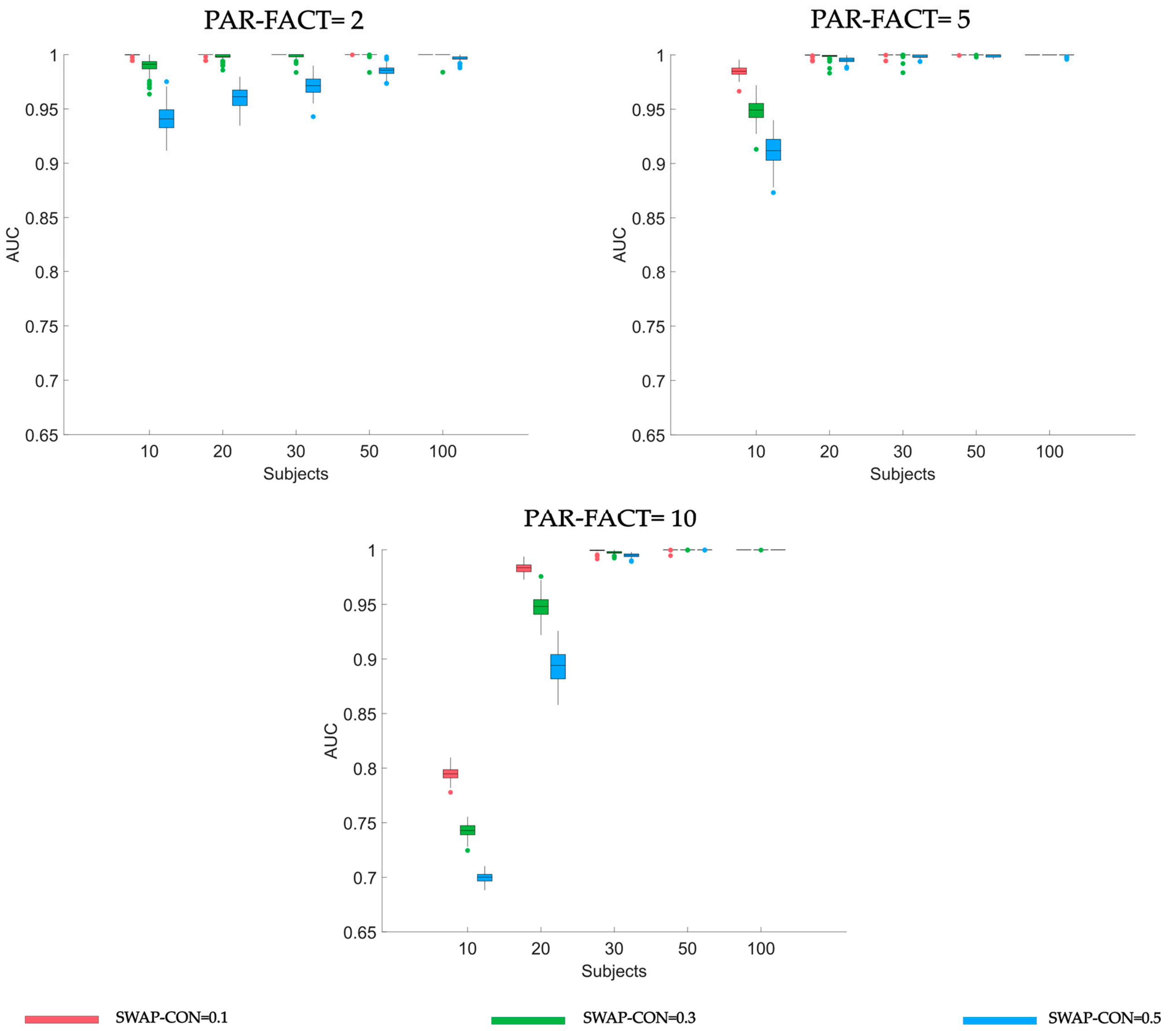

Figure S6. Boxplot diagrams reporting AUC distributions obtained applying PARAFAC algorithm using three different numbers of PAR-FACT (panels along rows in the figure) on synthetic data generated using five different levels of SAMPLE-SIZE (along x-axis), different levels of SWAP-CON (different colors of the bars). Results refer to synthetic datasets of dimension equal to 50 nodes.
